# Biotransformation of Daidzein, Genistein, and Naringenin by *Streptomyces* Species Isolated from High-Altitude Soil of Nepal

**DOI:** 10.1155/2021/9948738

**Published:** 2021-06-19

**Authors:** Lasata Shrestha, Bishnu P. Marasini, Suman Prakash Pradhan, Rajib Kumar Shrestha, Suraj Shrestha, Kamal Prasad Regmi, Bishnu Prasad Pandey

**Affiliations:** ^1^Department of Chemical Science and Engineering, Kathmandu University, Kathmandu, Nepal; ^2^Department of Biotechnology, National College, Tribhuvan University, Naya Bazar, Kathmandu, Nepal; ^3^Department of Environmental Science and Engineering, Kathmandu University, Kathmandu, Nepal; ^4^Aquatic Ecology Centre, Kathmandu University, Kathmandu, Nepal; ^5^Department of Food Technology and Quality Control, Ministry of Agriculture and Livestock Development, Babarmahal, Kathmandu, Nepal

## Abstract

Flavonoids have achieved widespread importance in pharmaceutical, food, and cosmetics industries. Furthermore, modification of these naturally occurring flavonoids to structurally diverse compounds through whole cell biotransformation with enhanced biological activities has numerous biotechnological applications. The present study investigated the biotransformation potential of *Streptomyces* species isolated from a high-altitude-soil sample towards selected flavonoid molecules. The biotransformed metabolites were confirmed by comparing the HPLC chromatogram with authentic compounds and LC-MS/MS analysis. Of these isolates, *Streptomyces* species G-18 (Accession number: MW663767.1) catalyzed isoflavone molecules daidzein and genistein to produce hydroxylated products at 24 h of reaction condition in a whole cell system. The hydroxylation of daidzein (4′,7-dihydroxyisoflavone) was confirmed at 3′-position of the B ring to produce 3′,4′,7-trihydroxyisoflavone. In addition, *Streptomyces* species G-14 (Accession number: MW663770.1) and *Streptomyces* species S4L (Accession number: MW663769.1) also revealed the transformation of daidzein (4′,7-dihydroxyisoflavone) to hydroxy daidzein at a distinct position than that of G-18 isolates, whereas thee *Streptomyces* species S4L reaction mixture with naringenin as a substrate also revealed the hydroxylated product. Our results demonstrated that microorganisms isolated from different ecological niches have broad application.

## 1. Introduction

*Streptomyces* are Gram-positive bacteria known for their production of diverse groups of secondary metabolites; many of which are now commercially used as antibiotics [1]. Over the years, these microorganisms evolved ecological roles by involving in changing soil dynamics, pathogenesis, nutrients exhaustions, and various abiotic and biotic stresses as well [2]. The secondary metabolites produced by *Streptomyces* provide the organism with competitive advantages to cope with the changing environment [3]. Genome analysis revealed that genes involved in biosynthesis of secondary metabolites are mostly clustered and their expression is highly regulated by different regulatory enzymes. *Streptomyces* are not only known for their production of diverse secondary metabolites but also used as a host for the production of foreign molecules through various biochemical engineering processes [4].

The unique metabolic diversity and enzymatic capabilities of *Streptomyces* make them a robust host for the production of diverse chemical constituents. *Streptomyces griseus* catalyzes a wide variety of biotransformation reactions towards flavonoids [5]. The majority of *S. griseus* catalytic reactions include O- and N-dealkylations, hydroxylation, epoxidation, and carbon bond fission [6, 7]. Microbial biotransformations provide the necessary precursors for the production of new compounds [8]. In addition, microorganisms have tendencies to modify functional groups of secondary metabolites to enhance their biological activities. Daidzein, a soy isoflavone, was hydroxylated by *Aspergillus oryaze* to form 6-hydroxydadizein and 8-hydroxydaidzein [9]. Moreover, this hydroxylated daidzein has ten-fold increase in antityrosinase activity compared with the precursor compounds of daidzein [10]. Hence, identification of a new microorganism with robust potential to convert foreign molecules is of scientific interest.

Flavonoids are a diverse group of phytochemicals that are produced by various plants species and play an important role in plant growth and development, as well as to defend against microorganisms and pests serving as means of plant-animal warfare [11]. Although plants are the potential source of flavonoids, many these flavonoids can be produced by the microorganisms through the whole cell biotransformation systems or through metabolic pathway engineering [12]. Interestingly, flavonoids that are not produced by plants can be produced by microbes through specific enzymatic modification [13]. Many microorganisms can perform several biochemical reactions such as hydroxylation, methylation, and glycosylation through the specific set of enzymes [14]. In addition, the advantage of using the whole cell biotransformation systems is that the microbes produce the necessary cofactors needed for the biochemical reactions. Furthermore, microorganisms isolated from different climatic and geographic conditions are proven to be effective for the production of diverse enzymes with different catalytic properties, which in turn play important roles in the production of diverse secondary metabolites. Hence, this study was designed in such a way to evaluate the biotransformation of flavonoid compounds daidzein, genistein, and naringenin by *Streptomyces* species isolated from a high-altitude-soil sample.

## 2. Materials and Methods

### 2.1. Chemicals

HPLC-grade methanol, water, and acetonitrile were purchased from Fisher Scientific, India. Genistein (4,5,7-trihydroxyisoflavone), chrysin (5,7-dihydroxyflavone), and apigenin (4,5,7-trihydroxyflavone), naringenin, quercetin, daidzein, and 3′,4′,7-trihydroxyisoflavone were provided by Prof. Byung Gee Kim, Seoul National University, South Korea.

### 2.2. Soil Sample Collection and Species Isolation

The soil samples for the isolation of *Streptomyces* species were collected from Gosaikunda, Langtang National Park, Nepal, 4380 masl (28.0820° N, 85.4150° E). The *Streptomyces* species were isolated in an ISP2 medium with the serial dilution method using a sterile spreader. All strains were characterized by their Gram staining, growth patterns, and colony morphology.

### 2.3. *Streptomyces* Culture Medium

Isolated *Streptomyces* species were cultivated in an R2YE medium containing 10.3% sucrose, 1% glucose, 1% MgCl_2_·6H_2_O, 0.025% K_2_SO_4_, 0.5% yeast extract, 0.01% casamino acid, 0.57% TES, 0.005% K_2_HPO_4_, 0.03% CaCl_2_·2H_2_O, 0.003% L-proline, 2 mL of trace element solution, and 5 mL of 1 N NaOH. The trace elements solution contained 0.004% ZnCl_2_, 0.02% FeCl_3_·6H_2_O, 0.001% CuCl_2_·2H_2_O, 0.001% MnCl_2_·4H_2_O, and 0.001% Na_2_B_4_O_7_·10H_2_O.

### 2.4. Growth Analysis

The growth curve of isolated *Streptomyces* species (G-10, G-14, G-18, and S4L) was investigated by growing them for 96 h in 50 mL of the R2YE medium, inoculated individually using 1 mL of preculture inoculums of each species. The isolates were incubated on a rotary shaker (250 rpm) at 28°C for four days. An aliquot of 1 mL of the culture broth was withdrawn at 12 h interval, from 0 h to 93 h. The cell mass was harvested at 12,000 rpm for 5 min, and the supernatant was discarded. The pellets were washed with distilled water and dried in oven at 70°C. Dry weight of cell mass was measured using a weighing machine, and graphs were plotted as dry cell weight *vs.* time.

### 2.5. Whole Cell Biotransformation

Whole cell biotransformation of flavonoids using *Streptomyces* species was carried out in the R2YE medium. Flavonoid substrate molecules were individually dissolved in dimethyl sulfoxide, and 100 mM of stock solution of each substrate was prepared. *Streptomyces* species were grown in the R2YE medium using the preculture inoculums. 100 *μ*M of substrate molecules daidzein, genistein, naringenin, and quercetin were fed into 72 h culture broth of *Streptomyces* species. The progresses of the biotransformation reactions were monitored after 24 h of reaction. Metabolites were extracted using ethylacetate as an extracting solvent. The organic layer was dried using a vacuum evaporator.

### 2.6. Analytical Methods

The metabolites were analyzed by High-Performance Liquid Chromatography (LC-2030 Shimadzu, Kyoto, Japan) equipped with a UV detector and C18 column (4.6 × 150 mm, 5 *μ*m particle size). The flow rate of the solvent was 1.0 mL/min, and the injection volume was 10 *μ*L. The mobile phase used for the analysis was acetonitrile (CH_3_CN): water (0.1% TFA) = 3 : 7 (v/v). The UV detection was carried out at 254 nm. The mass spectrometry was performed with an Agilent Jet Stream Electrospray Ionization source (AJS ESI) in MS2 scan and positive polarity mode. Acetonitrile and 0.1% formic acid in water in the ratio of 3 : 7 were used throughout the run. The flow rate was 0.5 mL/min. The column temperature was 30°C, and injection volume was 2 *μ*L.

## 3. Results

### 3.1. Growth Curve Analysis of *Streptomyces* Species

**Figure 1:**
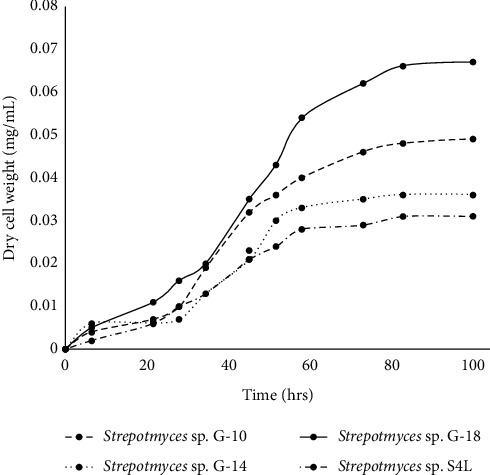
Growth curves of the *Streptomyces* species (G-10, G-14, G-18, and S4L) in the R2YE medium.

Prior to the biotransformation of flavonoids, the growth pattern of isolated *Streptomyces* species was evaluated. These isolates G-10, G-14, G-18, and S4L were grown in the R2YE medium for 96 h at 28°C. The *Streptomyces* species grew exponentially until 68 hours in the R2YE medium (Figure 1). The highest dry weight biomass (mg/mL) was recorded by the incubation of 20 h to 48 h of period, which revealed the exponential growth phase. However, from 48 h onward, the growth was stagnant, and no significant increase in biomass was observed. Among the analyzed *Streptomyces* species, the highest growth was observed in *Streptomyces* species G-18, followed by *Streptomyces* species G-10.

### 3.2. Biotransformation of Flavonoids

The whole cell biotransformation reaction with isolated *Streptomyces* species was carried out with flavonoids as substrate molecules. Analysis of the reaction products was carried out using HPLC equipped with a UV detector. Preliminary analysis revealed that, among several flavonoid molecules screened for the biotransformation potential by *Streptomyces* species, G-18 and G14 showed the biotransformed products toward daidzein as a substrate. Furthermore, G-18 also revealed the biotransformation product towards genistein, whereas S4L showed the biotransformation potential towards naringenin as a substrate. However, all other evaluated substrates did not reveal any product formation in our preliminary investigation (Table 1).

**Figure 2:**
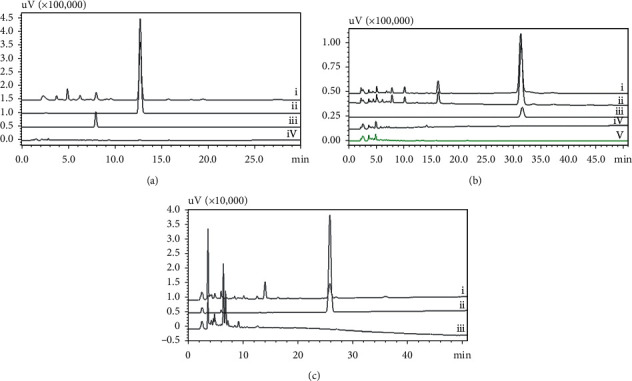
(a) HPLC chromatogram of biotransformation of daidzein, where (i) *Streptomyces* species G-18 with daidzein, (ii) daidzein, (iii) 7,3′,4′-trihydroxyisoflavone, and (iv) *Streptomyces* species G-18 without daidzein. (b) HPLC chromatogram of biotransformation of genistein, where (i) *Streptomyces* species G-14 with genistein, (ii) *Streptomyces* species G-18 with genistein, (iii) genistein, (iv) *Streptomyces* species G-18 without genistein, and (v) *Streptomyces* species G-14 without Genistein. (c) HPLC chromatogram of biotransformation of naringenin, where (i) *Streptomyces* species S4L with naringenin, (ii) naringenin, and (iii) *Streptomyces* species S4L without naringenin.

**Figure 3:**
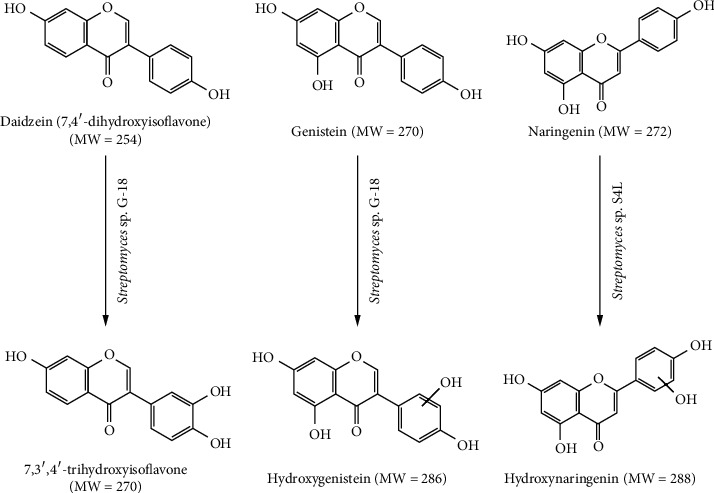
Schematic representation of biotransformation of daidzein, naringenin, and genistein by isolated *Streptomyces* species.

Further confirmation of the reaction metabolites was carried out by comparison of the HPLC chromatogram with authentic compounds. The substrate and reaction products were clearly separated. The reaction product of daidzein was matched completely with the authentic 7,3′,4′-trihydroxyisoflavone. Hence, daidzein biotransformation by *Streptomyces* species G-18 was at the 3′-position (Figure 2(a))), whereas *Streptomyces* species S4L and G-14 had a slight shift in the product peak, and this could be due to the hydroxylation of daidzein at 6- and 8-position (see supplementary data (available here)), whereas with genistein, the product peak was distinct from genistein, as observed by comparision of the HPLC chromatogram of authentic genistein, reaction product of *Streptomyces* species G-18, and control-only *Streptomyces* species G-18 without substrate (Figure 2(b)). Moreover, reaction products of *Streptomyces* species S4L show distinct product peaks with naringenin as a substrate in comparison of the HPLC chromatogram with the control reactions (Figure 2(c)). The product identification and characterization of biotransformation products were performed by mass spectrometry analysis. A schematic representation of the biotransformation by the isolated species is shown in Figure 3.

### 3.3. Mass Spectrometry Analysis of Metabolites

**Figure 4:**
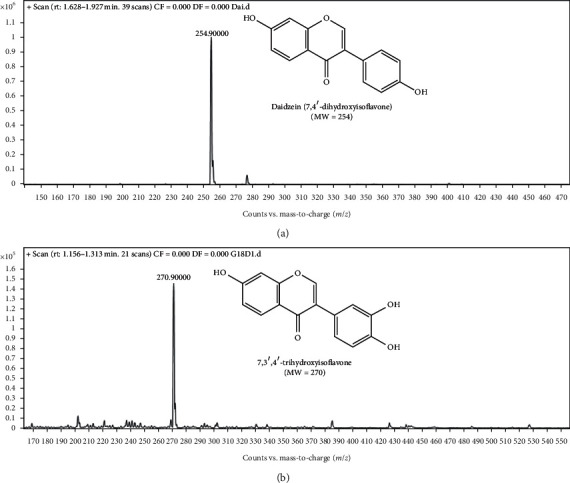
Mass spectra of daidzein and its products by *Streptomyces* species G-18.

Further confirmation of the biotransformation products was carried out by mass spectrometry analysis. The reaction mixture of *Streptomyces* sp. G18 with daidzein showed two distinct MS peaks [M + H]^+^ = 254.9 corresponding to daidzein and [M + H]^+^ = 270.9 product. The increase of molecular mass by 16 units is due to the addition of one hydroxy group in daidzein. Based on the comparative HPLC chromatogram with authentic 3′,7,4′-trihydroxyisoflavone and further analysis by mass spectrometry, we confirm that the daidzein was biotransformed by *Streptomyces* species G-18 to the 3′-position (Figure 4).

**Figure 5:**
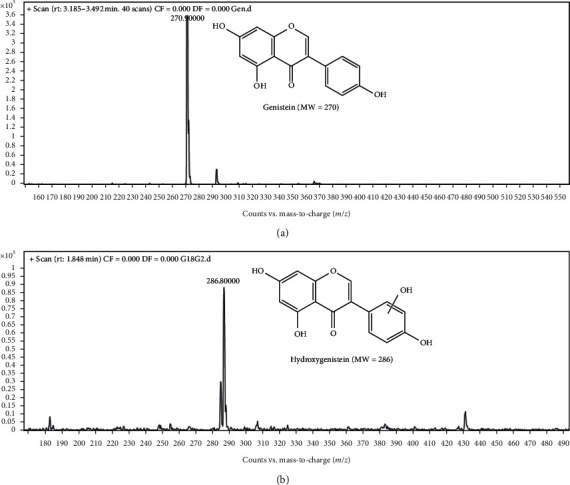
Mass spectra of genistein and its products by *Streptomyces* species G-18.

**Figure 6:**
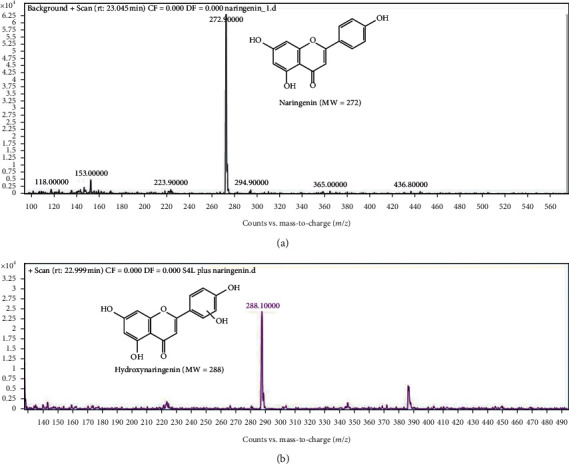
Mass spectra of naringenin and its products by *Streptomyces* species S4L.

Furthermore, genistein biotransformation by *Streptomyces* species G-18 was confirmed by mass spectrometry. MS spectra of genistein [M + H]^+^ = 270.9 and hydroxylated genistein [M + H]^+^ = 286.8 revealed the increase of the mass by 16 units in the reaction metabolites, which is hydroxygenistein (Figure 5). Based on the MS spectra, we speculate that the genistein must be hydroxylated at the 3′-position, whereas *Streptomyces* species S4L metabolized the naringenin (*m*/*z* = 272) to hydroxylated naringenin (*m*/*z* = 288), evident by the increase in the product peak by 16 units compared to naringenin (Figure 6).

## 4. Discussion

Soy isoflavones are naturally occurring phytochemicals that are associated with diverse health benefits [15]. In addition, they are biotransformed through oxidative and reductive reaction in diverse microorganism, significantly increasing the biological activities compared to original substrates [16]. Recently, food, cosmetics, and nutraceutical industries have shown interest in the functional derivatives of the isoflavones and flavones. Therefore, scientists all around the world are trying to understand the biosynthetic pathways for the production of valuable bioactive compounds using whole cell or enzymatic biotransformation. Esaki et al. have reported that *Aspergillus saitoi* from fermented food have shown to have tendencies to produce the 8-ortho hydroxydaidzein and 8-ortho hydroxygenistein [17]. Since then, the possibilities of microbial whole cell/enzymatic biotransformation emerged. Komiyama et al. have also reported the hydroxylation of the isoflavone product by *Streptomyces* species OH-1094 [18]. Furthermore, Sandra et al. have reported the fungal strains, namely, *Beauveria bassiana* AM 278, *Absidia glauca* AM 177, and *Absidia coerulea* AM 93, were capable of glycosylation of daidzein, genistein, and biochanin A [19].

Although plants are the source of the flavonoids, extraction and purification do not meet the industrial demands. Moreover, many nonnatural flavonoid molecules can be produced using a microbial host. Choi et al. have reported that O-methyltransferase from *Streptomyces avermitilis* involved in the production of 4′,7-dihydroxy-3′-methoxyisoflavone, a nonnatural compound [20]. Hence, microbial production of biotransformed molecules using whole cell or enzymes is alternative. Recent advancement in genetic engineering has made it possible to manipulate genes and engineer the host microorganism as well for efficient production of nonnatural isoflavonoids in a microbial host. Among the known microbial hosts, *Streptomyces* has been proven to be an effective host for the production of diverse chemical compounds. The biosynthetic gene cluster of secondary metabolites producing *Streptomyces* sp. involves several enzymes, which might be effective for the biotransformation of diverse molecules. Furthermore, cofactors such as NADH and/or NADPH required for the enzymatic modification of the flavonoid molecules are produced endogenously with in the microbial host [21]. It has been reported that the overexpression of cytochrome p450 enzyme CYP105D7 with its redox partners FdxH and FprD in *S. avermitilis* host could produce 112.5 mg of 7,3′,4′-trihydroxyisoflavone [22, 23].

Despite the significant effort in the past for the production of ortho-hydroxylated daidzein using whole microbial cell or enzymatic reaction, industrial-scale production of ortho-dihyroxyisoflavone (ODI) is still a big challenge. The bioconversion of flavonoids and isoflavonoids by microorganisms is very low and also hindered by solubility of substrate molecules in an aqueous solution. Moreover, ortho-dihydroxylated metabolites undergoe rapid degradation by the dioxygenase enzymes. Hence, screening of robust microorganisms from different environments as well as a protein engineering approach can overcome such limitations. In our investigation, *Streptomyces* species G-18 revealed hydroxylated products of daidzein and genistein. Daidzein was hydroxylated at the 3′-position, which is confirmed by comparing the HPLC chromatogram of authentic compounds and mass spectrometry analysis. Furthermore, S4L and G-14 also hydroxylated daidzein at different positions than that of G-18 as confirmed by comparing the HPLC chromatogram. This clearly indicates that enzymes involved in daidzein biotransformation by these strains are different and highly regioselective. However, the bioconversion of daidzein to hydroxydaidzein by the stain S4L and G-14 is very low to purify the products for structure elucidation. Based on the position in the HPLC chromatogram, we speculate that hydroxylation took place at the 6- and 8-position of daidzein by strain S4L and G-14. On the other hand, strain S4L also revealed the biotransformation potential toward naringenin. Mass spectrometry analysis revealed the increase in mass by 16 units; hence, the product is hydroxylated naringenin. The hydroxylated product of naringenin might be eriodictyol; as reported in cases of microbial biotransformation, the product of naringenin is eriodictyol [24]. Further optimization of the reaction products might increase the production of these hydroxylated metabolites, which enable for structure identification using NMR.

## 5. Conclusions

Streptomyces species are well known for production of diverse secondary metabolites and proven to be the effective host for the production of diverse compounds. However, *Streptomyces* species isolated from the high-altitude-soil sample have not been explored for biotransformation potential towards flavonoids. High altitude with a cold environment could be the source of unique biodiversity. In the present study, we have successfully demonstrated that *Streptomyces* species isolated from high-altitude-soil samples are effective for the biotransformation of daidzein (4′,7-dihydroxyisoflavone) to 3′,4′,7-trihydroxyisoflavone and genistein (4′,5,7-trihydroxyisoflavone) to hydroxygenistein. Furthermore, isolated species also revealed the biotransformation potential towards naringenin to hydroxynaringenin. Furthermore, identification of the specific enzymes from the isolated species as well as host engineering for the efficient production of the hydroxylated isoflavonoid and flavonoid molecules has tremendous industrial importance. Moreover, our results open up the possibilities in future for the search of a novel biocatalyst from diverse ecological niches.

## Figures and Tables

**Table 1:** Screening of flavone, isoflavone, and flavanone against *Streptomyces* species for biotransformation of diverse substrate molecules. Analysis was based on the HPLC chromatogram of unknown peaks, where (+) means biotransformation positive results and means no biotransformation based on the unknown peak in the HPLC chromatogram.

Substrate	*Streptomyces* sp. S4L	*Streptomyces* sp. G10	*Streptomyces* sp. G14	*Streptomyces* sp. G18
Flavone	−	−	−	−
Flavanone	−	−	−	−
3-Hydroxyflavone	−	−	−	−
7-Hydroxyflavone	−	−	−	−
Daidzein	+	−	+	+
Genistein	−	−	+	+
Apigenin	−	−	−	−
Chrysin	−	−	−	−
Naringenin	+	−	−	−
Quercetin		+	+	+

## Data Availability

The datasets used and/or analyzed during the current research are available from the corresponding author on reasonable request.

## Supplementary Materials

Supplementary Materials Supplementary Figure S1: HPLC chromatogram of biotransformation of *Streptomyces* sp. G-14 with daidzein. (i) *Streptomyces* sp. G-14 with daidzein as a substrate, (ii) daidzein only, and (iii) Streptomyces sp. G-14 crude extract without daidzein. Supplementary Figure S2: HPLC chromatogram of biotransformation of S4L with daidzien. (i) *Streptomyces* sp. S4L with daidzein as a substrate, (ii) daidzein only, and (iii) Streptomyces sp. S4L crude extract without daidzein. Supplementary Figure S3: HPLC chromatogram of biotransformation of quercetin, where (i) *Streptomyces* sp. G-14 with quercetin, (ii) quercetin, and (iii) *Streptomyces* Sp. G-14 crude extract without quercetin.
