# Supplementary material for: Biotransformation of Daidzein, Genistein, and Naringenin by Streptomyces Species Isolated from High-Altitude Soil of Nepal
Source: Int J Microbiol. 2021 Jun 19;2021:9948738. doi: 10.1155/2021/9948738 (PMC8238566; doi:10.1155/2021/9948738)
Supplement: Supplementary Materials — Supplementary Figure S1: HPLC chromatogram of biotransformation of Streptomyces sp. G-14 with daidzein. (i) Streptomyces sp. G-14 with daidzein as a substrate, (ii) daidzein only, and (iii) Streptomyces sp. G-14 crude extract without daidzein. Supplementary Figure S2: HPLC chromatogram of biotransformation of S4L with daidzien. (i) Streptomyces sp. S4L with daidzein as a substrate, (ii) daidzein only, and (iii) Streptomyces sp. S4L crude extract without daidzein. Supplementary Figure S3: HPLC chromatogram of biotransformation of quercetin, where (i) Streptomyces sp. G-14 with quercetin, (ii) quercetin, and (iii) Streptomyces Sp. G-14 crude extract without quercetin. [file 9948738.f1.doc]

**Supplementary data**


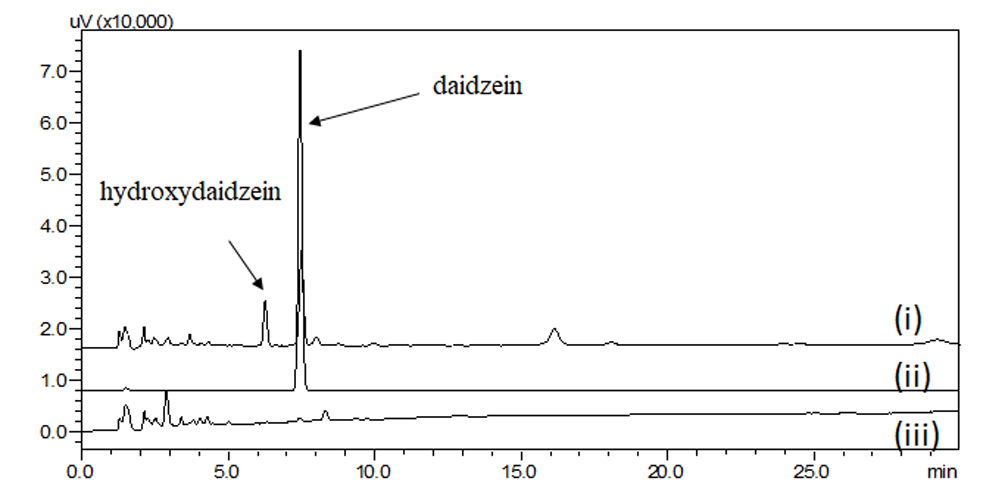


**Figure S1**. HPLC chromatogram of biotransformation of Streptomyces sp. G-14 with daidzein. (i) Streptomyces sp. G-14 with daidzein as a substrate (ii) daidzein only (iii) Streptomyces sp. G-14 crude extract without daidzein


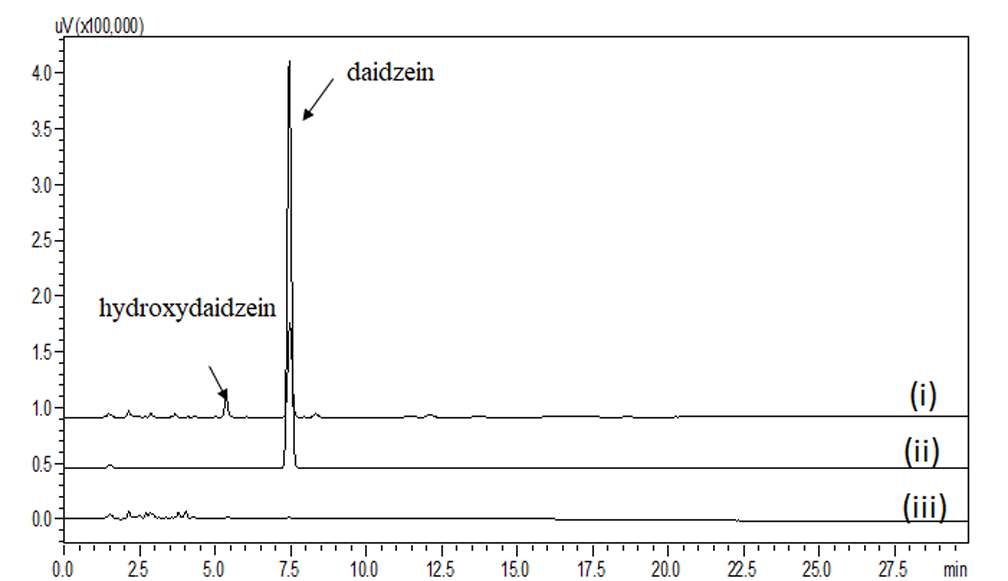


**Figure S2**. HPLC chromatogram of biotransformation of S4L with daidzien. (i) Streptomyces sp. S4L with daidzein as a substrate (ii) daidzein only (iii) Streptomyces sp. S4L crude extract without daidzein


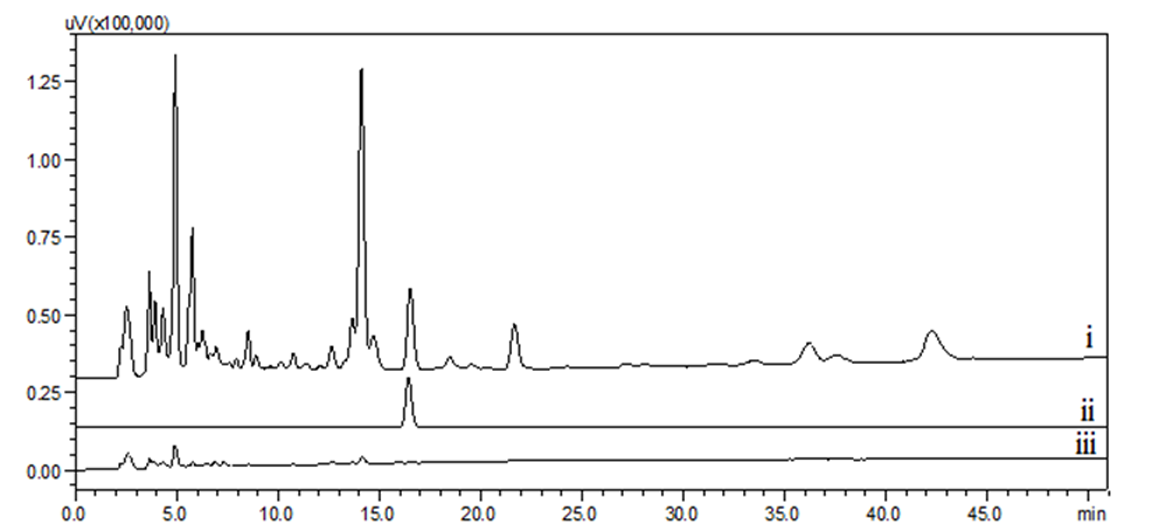


**Figure S3**. HPLC chromatogram of biotransformation of quercetin. Where, (i) Streptomyces sp. G-14 with quercetin (ii) quercetin and (iii) Streptomyces Sp. G-14 crude extract without quercetin
